# Credentialed pharmacist-led home medicines reviews targeting treatable traits and their impact on health outcomes in people with chronic obstructive pulmonary disease: a pre- and post-intervention study

**DOI:** 10.1007/s11096-024-01819-6

**Published:** 2024-10-28

**Authors:** Muhammad Rehan Sarwar, Vanessa Marie McDonald, Michael J. Abramson, Sally Wilson, Anne E. Holland, Billie Bonevski, Ajay Mahal, Eldho Paul, Brian Meier, Johnson George

**Affiliations:** 1 Centre for Medicine Use and Safety, Faculty of Pharmacy and Pharmaceutical Sciences, Monash University, Parkville, VIC Australia; 2 Centre for Research Excellence in Severe Asthma and Centre of Excellence in Treatable Traits, National Health and Medical Research Council, The University of Newcastle, Newcastle, NSW Australia; 3 The Priority Research Centre for Healthy Lungs, School of Nursing and Midwifery, Newcastle, NSW Australia; 4 Department of Respiratory and Sleep Medicine, John Hunter Hospital, Hunter Medical Research Institute, Newcastle, NSW Australia; 5 School of Public Health and Preventive Medicine, Monash University, Melbourne, VIC Australia; 6 School of Translational Medicine, Monash University, Melbourne, VIC Australia; 7 College of Medicine and Public Health, Flinders Health and Medical Research Institute, Flinders University, Bedford Park, SA Australia; 8 The Nossal Institute for Global Health, The University of Melbourne, Melbourne, VIC Australia

**Keywords:** Chronic obstructive pulmonary disease, Home medicines review, Medication adherence, Pharmacists, Treatable traits

## Abstract

**Background:**

Patients with chronic obstructive pulmonary disease (COPD) should engage in self-management strategies targeting behavioural traits and lifestyle risk-factors for optimal outcomes.

**Aim:**

To evaluate the impact of credentialed pharmacist-led home medicines review (HMR) targeting treatable traits (TTs) on health outcomes in COPD in primary care.

**Method:**

A pre- and post-intervention study was nested within a cluster-randomised controlled trial. A total of 81 participants with COPD from 21 Australian general practices received an HMR with a credentialed pharmacist targeting TTs. Changes in health outcomes at 6 and 12 months from baseline were assessed.

**Results:**

Ten TTs were assessed and targeted during the HMR. At baseline, no-one had a written action plan for managing exacerbations, and medication adherence was sub-optimal in 85% of patients. Additionally, 53% of participants demonstrated inadequate inhaler device technique, while 52% were current smokers. At 6-months follow-up, significant improvements were observed in health-related quality of life (St. George’s Respiratory Questionnaire score = 34.6 versus 39.1 at baseline, *p* = 0.006), health status (COPD Assessment Test score = 12 versus 16, *p* = 0.002), anxiety (Hospital Anxiety and Depression Scale (HADS)–Anxiety score = 2.0 versus 5.0, *p* < 0.001), depression (HADS-Depression score = 1.0 versus 5.0, *p* < 0.001), self-reported smoking (47% versus 51.9%, *p* = 0.031) and treatment adherence (Tool for Adherence Behaviour Screening score = 12.5 versus 10.0, *p* = 0.002). At 12-months: health status, anxiety, depression, smoking abstinence and adherence to treatment, continued to show statistically significant improvements compared to baseline measurements.

**Conclusion:**

HMRs targeting TTs improved health outcomes in people with COPD. Credentialed pharmacists in primary care can work alongside general practitioners to optimise COPD management.

**Supplementary Information:**

The online version contains supplementary material available at 10.1007/s11096-024-01819-6.

## Impact statements

- Targeting treatable traits through home medicines reviews (HMRs) can lead to significant improvements in health status, smoking abstinence, and treatment adherence, and may be an accessible model of care to address the complexity of COPD in the community.
- HMRs conducted in a structured collaborative manner can provide valuable support to the patients as well as their primary health professionals, particularly general practitioners.
- The study provides evidence for incorporating HMRs into clinical practice guidelines, supporting the integration of pharmacist-led reviews for better patient outcomes in COPD.

## Introduction

Chronic obstructive pulmonary disease (COPD), a complex and heterogeneous disease, is the third leading cause of death worldwide [1]. In 2016, 251 million cases of COPD were recorded worldwide, according to estimates from the Global Burden of Disease (GBD) study [2]. It is estimated that by 2050, the number of COPD cases among individuals aged 25 and older could rise to 600 million globally [3].

Management of COPD is complex and requires people with COPD to engage in effective self-management to address behavioural treatable traits (TTs), and lifestyle risk factors, such as smoking, inadequate inhaler device technique, and sub-optimal treatment adherence [4]. Multiple co-morbidities are common among people with COPD, and they are often prescribed complex medication regimens, placing them at high risk for medication misadventure [5].

Sub-optimal adherence to treatment is a major contributor to emergency hospitalisation among COPD patients [5]. Optimal inhaler technique and medication adherence play a crucial role in reducing the risk of death and hospital admissions [6]. However, the rate of adherence to COPD medications has been found to be substantially lower in comparison to other chronic conditions, such as hypertension, diabetes, hyperlipidaemia and depression [7, 8]. Consequently, sub-optimal adherence to COPD medications was strongly associated with increased respiratory symptoms, mortality, hospitalisation, medical costs and decreased health-related quality of life [6, 9, 10]. Therefore, improving patient adherence to treatment is a top priority in all COPD intervention programs [10].

Health professionals’ communication style with patients can impact patients’ ability and tendency to adhere to their medications [11]. Providing adequate information about medications to patients (e.g. indications for medication, clear instructions on medication use, checking inhaler technique, knowledge about their medication) improves adherence [12, 13]. Providing tailored educational counselling raises the level of patient confidence, self-efficacy, and improves understanding of how to use their medications [14]. The literature supports the role of pharmacists in educating and counselling patients with COPD [15–17]. As medicines experts, pharmacists are ideally positioned to help patients better understand their clinical condition(s) and educate patients on how to take or use their medicines.

Management of COPD in primary care is an ongoing challenge in Australia and is likely to become more difficult with the rapidly increasing number of people with COPD and widespread shortages of general practitioners (GPs) [18].

A home medicines review (HMR) is a clinical process that considers the patient’s medicines and health in order to enhance the quality use of medicines (QUM) and reduce the number of adverse medicines events [19]. An HMR is a collaborative process between the referring medical practitioner (‘referrer’), the GP (if this is not the referrer), other members of the oatient’s healthcare team (including the patient’s usual community pharmacy if they have one), accredited pharmacist (credentialed pharmacist), patient, and where appropriate, a carer.

The potential role of credentialed pharmacists targeting TTs in the management of COPD in primary care has been largely unexplored. Home visits provide an ideal environment for brief interventions covering smoking cessation, medication management and health behaviour change, followed by reinforcement of these messages through the patient’s GP.

### Aim

Our study aimed to evaluate the impact of credentialed pharmacist-led HMR targeting TTs on health outcomes in people with COPD. We hypothesised that credentialed pharmacist-led HMR targeting TTs will improve health outcomes in people with COPD.

### Ethics approval

All participants gave written informed consent at the time of enrolment. Monash University Human Research Ethics Committee approved these secondary analyses of the data (MUHREC ID: 30353, dated: 25/08/2021).

## Method

### Design and study population

This was a secondary analysis of the Review of Airway Dysfunction and Interdisciplinary Community-based care of Adult Long-term Smokers (RADICALS) study. RADICALS was a two arm, cluster randomised controlled trial (cRCT) that tested an interdisciplinary model of care involving GPs, practice nurses, pharmacists, and physiotherapists in Australian general practices between March 2015 and January 2018. The study has been described in detail, and the primary results reported elsewhere [18]. COPD was confirmed by pre- and post-bronchodilator spirometry conducted in accordance with the American Thoracic Society/ European Respiratory Society (ATS/ERS) guidelines [20]. A total of 272 participants (Intervention = 157; Control = 115), with confirmed COPD were included in the trial and followed-up for 1 year. However, the uptake of all components of the RADICALS intervention was limited; only 31% (49 out of 157) completed the full intervention, and a quarter (26%, 41 out of 157) partially completed the intervention [21]. Of 157 participants in the intervention arm, 81 (51.6%) received an HMR, and were considered for the present analyses.

### Intervention

A comprehensive HMR was performed by a single credentialed consultant pharmacist (BM) to identify any medication-related problems and deviations from COPD-X guidelines [22]. HMR is a clinical and collaborative process between the referring medical practitioner, other members of the patient’s healthcare team (including the patient’s usual community pharmacy if they have one), credentialed pharmacist, patient, and where appropriate, a carer [19]. The HMR considers the patient’s medicines and health in order to enhance the QUM and reduce the number of adverse events. The patients were referred by their usual medical practitioner. A credentialled pharmacist in Australia is a pharmacist who has completed additional training and certification to offer specialised services, such as HMR. To achieve credentialing, pharmacists need to undertake advanced education, acquire relevant practical experience, and meet the standards established by the Australian Pharmacy Council (APC). Credentialing is managed by professional organisations of pharmacists such as the Pharmaceutical Society of Australia (PSA).

The pharmacist (BM) visited patients at their homes (a single 1.5 h visit), assessed medication adherence, inhaler techniques, and the patient’s knowledge about their medications, educated (verbally and using written information) on the role of medications in the management of COPD, management of comorbidities and behavioural risk factors, including smoking. If required, interventions for optimising medication adherence and inhaler use were also offered. After the initial interview the pharmacist prepared an HMR report for the patient’s referring GP that outlined their findings, including recommendations for optimising medication use and adherence to COPD-X guidelines [22]. The HMR report aimed to improve the referrer’s understanding of how their patient used their medicines and provided recommendations to assist the referrer and patient in developing a medication management plan. Additionally, an 8-week home-based pulmonary rehabilitation (HomeBase PR) programme [23] was delivered by a specifically trained physiotherapist, which comprised individually prescribed home-based aerobic and resistance exercise training. The uptake and timings of the HMRs and HomeBase PR varied, with the majority of HMRs conducted a few weeks after the initiation of the HomeBase PR.

### Data collection/measurements

Demographic data were collected at baseline. In addition, lung function (measured by pre- and post-bronchodilator spirometry), functional dyspnoea score (measured by modified Medical Research Council Dyspnoea Scale—mMRC) [24], depression and anxiety (measured by Hospital Anxiety and Depression Scale—HADS) scores [25], health status (measured by COPD Assessment Test—CAT) [26], comorbidities (measured by Charlson Comorbidity Index—CCI) [27], self-reported smoking status, medication adherence (measured by Tool for Adherence Behaviour Screening—TABS) [28], and Health Related Quality of Life (HRQoL, measured by St. George’s Respiratory Questionnaire—SGRQ) [29] were measured at baseline, 6 months and 12 months. Change in SGRQ total score at 6-months was the primary outcome.

### Treatable traits assessed and targeted by the credentialed consultant pharmacist

TTs of current smoking, inhaler device polypharmacy, inadequate inhaler device technique, overdosing or underdosing of prescribed medicines, under-treatment or lack of treatment, poor knowledge about therapy or condition, non-adherence, and exacerbation management were assessed and targeted by the credentialed consultant pharmacist. Details of the traits targeted along with their corresponding diagnostic criteria and individualised interventions are provided in Table 2.

### Statistical analyses

The Statistical Package for Social Sciences (SPSS for Windows Version 23.0. Armonk, NY: IBM Corp.) was used for data analyses. The distributions of continuous variables were assessed using Kolmogorov–Smirnov and Shapiro–Wilk tests. Baseline characteristics of participants were summarised using frequencies, percentages, means (standard deviations) or medians (interquartile ranges). Changes in health outcomes at 6 and 12 months from baseline were analysed using Wilcoxon signed rank test, paired-samples t- or McNemar Tests, as appropriate. A two-sided *p*-value < 0.05 was considered statistically significant.

Sensitivity analysis was performed using data from 76 participants in the intervention arm who did not receive a pharmacist-conducted HMR, although most did receive HomeBase PR. This analysis aimed to assess the impact of not receiving an HMR on the outcomes and to demonstrate the advantages of supplementary HMR within the context of receiving HomeBase PR. By comparing participants who received only HomeBase PR with those who received both HMR and HomeBase PR, this sensitivity analysis helps to isolate and understand the effect of the HMR alone.

Missing data could have affected the robustness of the analysis. Therefore, an additional sensitivity analysis was conducted using data obtained after imputing missing values to ensure the robustness of the primary analysis. Little’s Missing Completely at Random (MCAR) test was used to assess the nature of the missing data, and missing values were imputed using the Expectation–Maximization (EM) method.

## Results

Table 1 shows the baseline characteristics of participants who received the HMR. Most of the participants were male (59.3%) and their mean (±SD) age was 67.9 (±10.6) years. Most participants were born in Australia (75.3%) and had mild COPD (63.3%).

**Table 1:** Baseline characteristics of participants (N = 81)

Characteristic	
Age in years, mean (SD)	67.9 ± 10.6
Male, n (%)	48 (59.3)
Born in Australia, n (%)	61 (75.3)
Body mass index (BMI) kg/m^2^, median [IQR]	26.8 [23.3–31.4]
*mMRC Dyspnoea grade, n (%)*	
0	19 (23.5)
1	33 (40.7)
2	16 (19.8)
3	11 (13.6)
4	2 (2.5)
Heaviness of smoking index score (current smokers only), n (%)^*^	
Low nicotine dependence (score 0–2)	11 (26.8)
Moderate nicotine dependence (score 3–4)	22 (53.7)
High nicotine dependence (score 5–6)	8 (19.5)
Charlson comorbidity index, median [IQR]^†^	1.0 [1.0–1.0]
*Spirometry measurements (postbronchodilator)*	
FEV_1_ (litres), mean ± SD^‡^	1.79 ± 0.71
FVC (litres), median [IQR]^‡^	3.11 [2.56–4.65]
FEV_1_ / FVC, median [IQR]^‡^	0.59 [0.47–0.66]
FEV_1_% predicted, mean ± SD^‡^	65.1 ± 20.7
FVC % predicted, mean ± SD^§^	90.4 ± 19.0
*COPD severity*^*‡ ||*^*, n (%)*	
Mild	50 (63.3)
Moderate	16 (20.3)
Severe	13 (16.5)

^*^Missing data n = 1; †Missing data n = 58; ‡Missing data n = 2; §Missing data n = 3; || Severity of COPD based on COPD-X plan

IQR, interquartile range [25%le–75%le]; FEV_1_, forced expiratory volume in 1 s; FVC, forced vital capacity; mMRC, modified medical research council; PBD, post-bronchodilator; SD, standard deviation

Ten treatable traits (Table 2) were assessed and addressed with participants during the HMR. At baseline, none of the participants had a written action plan for managing exacerbations, and non-adherence was observed in 84.9% of participants. In addition, just over half of the participants demonstrated inadequate inhaler device technique and/or were identified as current smokers.

**Table 2:** Treatable traits assessed and targeted by the credentialed consultant pharmacist in the intervention group (n = 81)

Treatable trait	Indicator/measure	N (%)	Intervention
Current smoking	Self-reported current smoking or exhaled CO ≥ 10 ppm	42 (51.9)	Pharmacotherapy and smoking cessation counselling
Inhaler device polypharmacy	Number of inhaler devices ≥ 3	26 (32.1)	Minimising devices, combination therapy
Inadequate inhaler device technique*	Inhaler technique rating (adequate/inadequate)	35 (53.0)	Inhaler technique skills, change device type to suit patient abilities
Overdose prescribed	Home medicines review	1 (1.2)	Dose adjustment, dose frequency/schedule change
Underdose prescribed	Home medicines review	5 (6.2)	Dose adjustment, dose frequency/schedule change
Condition undertreated	Home medicines review	17 (21.0)	Guideline-based management
Condition untreated	Home medicines review	2 (2.5)	Guideline-based management
Confusion/poor knowledge about therapy or condition	Individual assessment during HMR	3 (3.7)	Counselling; self-management education
Non-adherence	Dispensing records/tool for adherence behaviour and screening (TABS^#^) score	62 (84.9)	Counselling; self-management education
Exacerbation management	Absence of self-management plan	81 (100)	Self-management education; written action plan

^*^Not applicable for (n = 15) participants. ^**#**^The TABS is a patient self-reported adherence measure, has two subscales—‘adherence’ (items 1–4) and ‘non-adherence’ (items 5–8)—each comprising four items to be answered on a 5-point likert-type scale (‘never’—1 to ‘always’—5). Total score on TABS = total for ‘adherence’—total for ‘non-adherence’. Good adherence: Differential of ≥ 15. Suboptimal adherence: differential of ≤ 14

At the 6-months follow-up: HRQoL (mean SGRQ score = 34.6 versus 39.1 at baseline), health status (median CAT score = 12 versus 16 at baseline), anxiety (median HADS-A score = 2.0 versus 5.0 at baseline), depression (median HADS-D score = 1.0 versus 5.0 at baseline), self-reported smoking abstinence (current smokers = 47% versus 51.9% at baseline) and adherence to treatment (median TABS score = 12.5 versus 10.0 at baseline) all showed statistically significant improvements (Table 3). Sensitivity analysis using data from those participants who did not receive a pharmacist-conducted HMR, at 6-months follow-up, did not show any statistically significant improvements in health outcomes, with the exception of HADS and adherence to treatment (Supplementary Table 1). Additional sensitivity analysis using data obtained after imputing missing values gave results similar to the primary analysis at the 6-months follow-up (Supplementary Table 2).

**Table 3:** Changes in health outcomes at 6 and 12 months from baseline

Outcomes	Baseline	6-months	6-months Vs baseline, *P-value*	12-months	12-months Vs baseline, *P-value*
SGRQ score, mean ± SD^*^	39.1 ± 16.6	34.6 ± 15.8	**0.006**	33.5 ± 20.3	0.077
CAT score, median [IQR]^†^	16.0 [8.0–20.0]	12.0 [8.0–16.5]	**0.002**	10.0 [6.0–16.5]	** < 0.001**
PBD FEV_1_%predicted, mean ± SD^‡^	64.5 ± 20.0	64.0 ± 20.6	0.656	73.5 ± 10.0	0.969
PBD FEV_1_ (litres), mean ± SD^‡^	1.79 ± 0.73	1.74 ± 0.73	**0.045**	2.01 ± 0.59	0.678
mMRC Dyspnoea score, median [IQR]^§^	1.0 [1.0–2.0]	1.0 [1.0–2.0]	0.693	1.0 [1.0–2.0]	0.889
HADS-A score, median [IQR]^||^	5.0 [3.0–9.0]	2.0 [0.0–5.0]	** < 0.001**	1.0 [0.0–4.0]	** < 0.001**
HADS-D score, median [IQR]^||^	5.0 [3.0–7.0]	1.0 [0.0–5.0]	** < 0.001**	1.0 [0.0–4.0]	** < 0.001**
Self-reported current smoking, n (%)	42 (51.9)	31 (47.0)	**0.031**	28 (45.9)	**0.016**
Adherence (TABS score), median [IQR]^**^	10.0 [7.0–13.0]	12.5 [9.0–14.0]	**0.002**	14.0 [9.0–15.0]	** < 0.001**

*p*-Values in bold are statistically significant (*p*<0.05)

^*^Missing data n = 3 at baseline, n = 16 at 6-months, n = 19 at 12-months; † missing data n = 12 at 6-months, n = 20 at 12-months; ‡ missing data n = 2 at baseline, n = 19 at 6-months, n = 75 at 12-months; § missing data n = 12 at 6-months, n = 19 at 12-months; || Missing data n = 1 at baseline, n = 12 at 6-months, n = 19 at 12-months; ¶ missing data n = 15 at 6-months, n = 20 at 12-months; ** Missing data n = 8 at baseline, n = 19 at 6-months, n = 23 at 12-months

CAT, COPD assessment test; HADS-A, hospital anxiety and depression scale—anxiety score; HADS-D, hospital anxiety and depression scale—depression score; IQR, interquartile range [25%le-75%le]; mMRC, modified medical research council; FEV_1_, forced expiratory volume in 1 s; FVC, forced vital capacity; PBD, post bronchodilator; SD, standard deviation; SGRQ, st george’s respiratory questionnaire; TABS, tool for adherence behaviour screening

At the 12-months follow-up: improvements in health status (median CAT score = 10.0 versus 16.0 at baseline), anxiety (median HADS-A score = 1.0 versus 5.0 at baseline), depression (median HADS-D score = 1.0 versus 5.0 at baseline), self-reported smoking abstinence (current smokers = 45.9% versus 51.9% at baseline) and adherence to treatment (median TABS score = 14.0 versus 10.0 at baseline) remained statistically significant (Table 3). Sensitivity analysis using data from participants who did not receive a pharmacist-conducted HMR, at 12-months follow-up, did not show statistically significant improvements in health outcomes with the exception of health status, HADS and adherence to treatment (Supplementary Table 1). Additional sensitivity analysis using data obtained after imputing missing values gave results similar to the primary analysis at the 12-months follow-up, except for the FEV_1_% predicted, which showed a statistically significant improvement (Supplementary Table 2).

## Discussion

This was the first study to evaluate the impact of credentialed pharmacist-led HMRs targeting TTs on health outcomes in people with COPD in primary care. Targeting TTs through HMRs resulted in clinically and statistically significant improvements in HRQoL, health status, smoking abstinence and adherence to treatment, while reducing anxiety and depression. This highlights a vital role for credentialed pharmacists in primary care alongside GPs, nurses, and other health professionals in the management of COPD. HMRs conducted in a structured collaborative manner can provide valuable support to the patients as well as their primary health professionals, particularly GPs.

At the 6-month follow-up, participants who received a pharmacist-conducted HMR demonstrated significant improvements in several key health outcomes compared to those who did not receive the HMR. These improvements highlight the positive impact of the pharmacist-conducted HMR intervention on various aspects of patient well-being and management. In contrast, sensitivity analysis of participants who did not receive the HMR revealed minimal statistically significant improvements, suggesting that the HMR had a substantial effect on these outcomes.

At the 12-month follow-up, the benefits of the HMR intervention persisted, with continued significant improvements in health status, anxiety, depression, smoking abstinence, and treatment adherence. The sensitivity analysis for the same period only showed limited improvements among those who did not receive the HMR. These results reinforce the efficacy of the pharmacist-conducted HMR in driving meaningful and sustained health improvements in patients with COPD.

The results showed better response with more health improvements at the 6-month follow-up compared to the 12-month follow-up. This could be due to several factors: (I) The initial impact of the intervention may have led to more immediate health improvements within the first 6 months. (II) The sustainability of behaviour changes may have declined, with fewer significant improvements by 12 months. (III) Participant engagement and adherence to the intervention may have decreased over time, affecting the outcomes at the 12-month mark.

Previous studies have suggested that pharmacist-led HMRs significantly improved health outcomes in people with chronic diseases in primary care [30–32]. A prospective study conducted among 412 community-dwelling older adults in China showed that HMRs were a practical means to optimise drug therapy (e.g., reduce drug-related problems [DRPs] and increase medication adherence) and improve patients’ HRQoL [31]. In an RCT conducted in Hobart, Australia, a 90-day follow-up by pharmacist for high-risk older people at home led to a decrease in DRPs and unplanned hospital readmissions [32]. Another RCT, conducted among 166 patients with Type 2 Diabetes Mellitus (T2DM) in Malaysia showed that at 6-months follow-up, HMRs significantly improved glycaemic control, medication adherence, QoL, and knowledge of T2DM, as well as reduced the number of DRPs and cost of medications wasted [30].

Emerging trials from tertiary care settings showed that pharmaceutical care has a positive impact on health outcomes in COPD. In an RCT involving 133 people with COPD in Jordan, structured education about COPD and symptom management delivered by clinical pharmacists significantly improved medication adherence, medication beliefs, patients’ COPD knowledge, and hospitalisation rates [5]. Another pharmaceutical care programme initiated in an emergency department in Spain, showed positive clinical benefits due to the reduced number and prevalence of drug-related negative outcomes [33]. In a pre- and post-intervention study in Vietnam, over a period of 12 months, the proportions of COPD patients with optimal medication adherence significantly increased from 37.4% to 53.2% through pharmacist-led care [34]. Furthermore, it demonstrated that individualized pharmaceutical care also improved HRQoL, inhalation technique and reduced readmissions in patients with COPD [35, 36].

Clinical pharmacists play a crucial role in the healthcare team by offering recommendations and interventions related to medication-related issues, particularly in the management of chronic conditions such as COPD [37]. In the Australian primary healthcare system, where GP time constraints limit patient education and counselling [38], utilising credentialed pharmacist expertise in optimising medicine use and promoting consumer self-management seems to be an optimal strategy. Recent research [16, 33, 34, 36] has demonstrated the benefits of integrating pharmacists into COPD management, revealing improvements in patient understanding, addressing drug-related issues, enhancing disease control, and reducing treatment costs. This highlights the positive impact of pharmaceutical care in patients with COPD, with pharmacists playing a crucial role in early detection through case finding, comprehensive medication management planning, and providing guidance on medication use, inhaler techniques, and adherence [39]. Our findings suggest that credentialed pharmacists are well-suited to enhance COPD management in primary care settings using a TT approach.

The Australian COPD-X Guidelines [22] and an international pulmonary rehabilitation (PR) statement [40] recommend referral to PR for all COPD patients, irrespective of disease severity. Participation in Homebase PR has been shown to improve HRQoL in patients with moderate to severe COPD [23]. Notably, our study sample predominantly comprised individuals with mild COPD and low levels of activity limitation, often self-reporting low mMRC grades. This may suggest a potential lack of recognition for the necessity of PR intervention in this subgroup. Consequently, there is a need for further research specifically targeting individuals with mild COPD to explore the impact of PR on diverse health outcomes and TTs based on a multidimensional assessment.

While treatable traits interventions are shown to be effective in improving multiple outcomes, the majority of studies have been conducted in tertiary care [41]. In a systematic review and meta-analysis involving 11 studies that targeted at least one TT in every TT domain (Pulmonary, Extra-Pulmonary, Behavioral/Risk-factors) only one study was identified in a primary care setting, and this was negative [41]. These data highlight the potential for a multidisciplinary TT model of care in the primary care setting. Such an approach is currently being tested in a cluster RCT evaluating the efficacy of a practice nurse-coordinated intervention targeting treatable traits in moderate-severe COPD in primary care, compared with usual care [42].

Our study has several strengths: to the best of our knowledge, it was the first study of credentialed pharmacist-led HMRs addressing TTs in people with COPD in primary care. These data were obtained from a pragmatic cluster-RCT, minimising selection bias, thus increasing the generalisability of the findings. We conducted a sensitivity analysis utilizing data exclusively from participants in the intervention group who did not undergo a pharmacist-conducted HMR, to ensure robustness of the primary analysis.

However, there were also some limitations: only a pre-post comparison could be performed, and seasonal variations and time were not controlled. No formal sample size calculation was performed for this secondary analysis. Data relating to exacerbations were not systematically captured in the RADICALS trial. Thus, we were unable to evaluate exacerbations as an important outcome of COPD. Being nested within the RADICALS trial, which was primarily designed to evaluate the efficacy of an interdisciplinary model of care for reducing the burden of smoking and COPD in Australian primary care settings before the TT concept emerged, traits were assessed retrospectively which was restricted by the information available from the trial data set. Thus, not all behavioural traits reported in the COPD literature could be assessed. Data were missing for some participants related to the outcome measurements, and therefore, an additional sensitivity analysis was conducted using data obtained after imputing missing values to ensure the robustness of the primary analysis. However, caution should be exercised when interpreting the findings. Future studies with more complete data are recommended to further validate these results.

## Conclusion

HMR by credentialed pharmacists could positively affect HRQoL, health status, anxiety, depression, smoking abstinence and adherence to treatment, in people with COPD. These results have important implications in managing COPD in the primary care setting. Future studies should explore strategies to optimise the delivery of TT-based interventions and evaluate their efficacy and cost effectiveness.

## Supplementary Information

Below is the link to the electronic supplementary material.

Supplementary file1 (DOCX 20 kb)
