# Supplementary material for: Credentialed pharmacist-led home medicines reviews targeting treatable traits and their impact on health outcomes in people with chronic obstructive pulmonary disease: a pre- and post-intervention study
Source: Int J Clin Pharm. 2024 Oct 28;47(1):157–65. doi: 10.1007/s11096-024-01819-6 (PMC11742330; doi:10.1007/s11096-024-01819-6)
Supplement: Supplementary file 1 — Supplementary file1 (DOCX 20 kb) [file 11096_2024_1819_MOESM1_ESM.docx]

**Supplementary Table 1: Changes in health outcomes at 6 and 12 months from baseline (Sensitivity Analysis** - **patients who did not receive HMR)**

| **Outcomes** | **Baseline** | **6-months** | **6-months Vs baseline,**  ***P-value*** | **12-months** | **12-months Vs baseline, *P-value*** |
| --- | --- | --- | --- | --- | --- |
| SGRQ score, mean (SD)^*^ | 28.5± 15.7 | 27.6 ± 17.99 | 0.617 | 67.4 ± 203.7 | 0.175 |
| CAT score, median (IQR)^†^ | 10.0 [6.0 ─ 13.5] | 9.0 [3.0 ─ 14.5] | 0.179 | 7.0 [3.75 ─ 12.0] | **0.031** |
| PBD FEV_1_% predicted, mean (SD)^‡^ | 76.4 ± 20.1 | 75.2 ± 20.9 | 0.315 | 91.7 ± 14.6 | 0.417 |
| PBD FEV_1_ (litres), mean [SD]^‡^ | 2.19 ± 0.75 | 2.16 ± 0.78 | 0.367 | 2.37 ± 0.16 | 0.658 |
| mMRC Dyspnoea score, median (IQR)^§^ | 1.0 [0.25 ─ 2.0] | 1.0 [0.0 ─ 2.0] | 0.545 | 1.0 [0.0 ─ 2.0] | 0.870 |
| HADS-A score, median (IQR)^\|\|^ | 6.0 [3.0 ─ 9.0] | 1.0 [0.0 ─ 5.0] | **<0.001** | 1.0 [0.0 ─ 4.0] | **<0.001** |
| HADS-D score, median (IQR)^\|\|^ | 4.0 [2.0 ─ 6.5] | 1.0 [0.0 ─ 3.0] | **<0.001** | 1.0 [0.0 ─ 3.0] | **<0.001** |
| Current smoking, n (% ) ^¶^ | 42 (55.3) | 24 (55.3) | 1.00 | 22 (46.8) | 1.00 |
| Adherence (TABS score), median [IQR]^**^ | 11.0 [8.0 ─ 14.0] | 12.0 [9.0 ─ 14.0] | **0.019** | 14.0 [9.75 ─ 15.25] | **<0.001** |
| *Missing data n = 3 at baseline, n = 35 at 6-months, n = 31 at 12-months; † Missing data n = 31 at 6-months, n = 26 at 12-months; ‡ Missing data n = 2 at baseline, n = 36 at 6-months, n = 73 at 12-months; § Missing data n = 31 at 6-months, n = 26 at 12-months; \|\| Missing data n = 3 at baseline, n = 31 at 6-months, n = 19 at 12-months; ¶ Missing data n = 33 at 6-months, n = 29 at 12-months; ** Missing data n = 14 at baseline, n = 41 at 6-months, n = 38 at 12-months.  **CAT:** COPD Assessment Test**; HADS-A:** Hospital Anxiety and Depression Scale – Anxiety score; **HADS-D:** Hospital Anxiety and Depression Scale – Depression score; **IQR** = Interquartile range [25%le-75%le]; **mMRC** = modified Medical Research Council; **FEV_1_** = Forced expiratory volume in 1 second; **FVC** = Forced vital capacity; **PBD**: Post bronchodilator; **SD**: Standard deviation; **SGRQ**: St George’s Respiratory Questionnaire; **TABS** = Tool for Adherence Behaviour and Screening | | | | | |

**Supplementary Table 2: Changes in health outcomes at 6 and 12 months from baseline (Sensitivity Analysis – Imputation of missing values)**

| **Outcomes** | **Baseline** | **6-months** | **6-months Vs baseline,**  ***P-value*** | **12-months** | **12-months Vs baseline, *P-value*** |
| --- | --- | --- | --- | --- | --- |
| SGRQ score, mean (SD) | 37.5± 17.1 | 33.5 ± 15.5 | **0.003** | 34.5 ± 19.97 | 0.168 |
| CAT score, median (IQR) | 16.0 [8.0 ─ 20.0] | 12.0 [8.0 ─ 16.5] | **0.004** | 10.0 [6.0 ─ 18.0] | **0.004** |
| PBD FEV_1_% predicted, mean (SD) | 64.96 ± 20.6 | 65.5 ± 21.2 | 0.581 | 71.4 ± 10.9 | **0.033** |
| PBD FEV_1_ (litres), mean [SD] | 1.79 ± 0.71 | 1.76 ± 0.71 | **0.048** | 1.94 ± 0.52 | 0.110 |
| mMRC Dyspnoea score, median (IQR) | 1.0 [1.0 ─ 2.0] | 1.0 [1.0 ─ 2.0] | 0.868 | 1.0 [1.0 ─ 2.0] | 0.819 |
| HADS-A score, median (IQR) | 5.0 [3.0 ─ 9.0] | 2.3 [0.2 ─ 5.0] | **<0.001** | 2.0 [0.0 ─ 4.5] | **<0.001** |
| HADS-D score, median (IQR) | 5.0 [3.0 ─ 7.0] | 1.0 [0.0 ─ 4.4] | **<0.001** | 1.9 [0.0 ─ 4.0] | **<0.001** |
| Current smoking, n (% ) | 42 (51.9) | 38 (46.9) | **0.006** | 39 (48.1) | **0.036** |
| Adherence (TABS score), median [IQR] | 9.86 [7.4 ─ 13.0] | 12.7 [9.2 ─ 14.0] | **<0.001** | 12.8 [9.0 ─ 14.9] | **<0.001** |
| **CAT:** COPD Assessment Test**; HADS-A:** Hospital Anxiety and Depression Scale – Anxiety score; **HADS-D:** Hospital Anxiety and Depression Scale – Depression score; **IQR** = Interquartile range [25%le-75%le]; **mMRC** = modified Medical Research Council; **FEV_1_** = Forced expiratory volume in 1 second; **FVC** = Forced vital capacity; **PBD**: Post bronchodilator; **SD**: Standard deviation; **SGRQ**: St George’s Respiratory Questionnaire; **TABS** = Tool for Adherence Behaviour and Screening | | | | | |
